# The renaissance of *Salmonella*-based cancer therapy: convergence of AI, synthetic circuits, and TME remodeling

**DOI:** 10.3389/fimmu.2026.1790215

**Published:** 2026-04-22

**Authors:** Zetian Zhang, Xingyu Jiang, Wenjun Huang, Xiaomeng Yang, Fan Li

**Affiliations:** 1 The Key Laboratory of Zoonosis, Department of Pathogen Biology, The Chinese Ministry of Education, College of Basic Medical Sciences, Jilin University, Changchun, China; 2 The Key Laboratory for Bionics Engineering, Ministry of Education, Jilin University, Changchun, China; 3 Engineering Research Center for Medical Biomaterials of Jilin Province, Jilin University, Changchun, China; 4 Key Laboratory for Health Biomedical Materials of Jilin Province, Jilin University, Changchun, China; 5 State Key Laboratory of Pathogenesis, Prevention and Treatment of High Incidence Diseases in Central Asia, Urumqi, Xinjiang, China

**Keywords:** artificial intelligence, attenuated *Salmonella*, bacterial cancer therapy, cancer immunotherapy, immunogenic cell death, synthetic biology, tumor microenvironment

## Abstract

Attenuated *Salmonella* has become a candidate bacterium for cancer immunotherapy, as it possesses tumor-targeting activity and is able to penetrate the hypoxic, immune-suppressive TME. However, preclinical successes were not successfully translated into early clinical trials (e.g., VNP20009) due to a critical “immunogenicity-safety trade-off,” in which the attenuation required to ensure safety compromised the ability to generate effective anti-tumor immunity. Here, we provide an updated perspective on the renaissance of the field and discuss how the integration of synthetic biology and artificial intelligence (AI) is overcoming these historical barriers, including AI-driven tools such as Cello and SHASI-ML. We outline the progression from static attenuation to dynamic, rational design, where programmable genetic circuits (e.g., synchronized lysis systems and hypoxia-responsive logic gates) enable bacteria to be engineered as intelligent microrobots that can be used for the safe delivery of therapeutics while inducing precise, on-target immune stimulation. In addition to cytotoxicity effects, we focus on how *Salmonella* reprograms the metabolic environment of the TME and discuss specific metabolic pathways, such as asparagine depletion-mediated T-cell paralysis, that contribute to rescuing exhausted effector cells. Finally, linking preclinical advances with recent clinical successes (e.g., Saltikva), we hypothesize that next-generation engineered *Salmonella* is more than just a vector but rather a full immunological modulator delivering the potential to convert “cold” tumors into “hot” environments capable of providing a synergistic platform to enhance checkpoint inhibitors and chemotherapeutics.

## 1 Introduction

In recent years, cancer immunotherapy has advanced substantially, shifting from nonspecific immune activation toward more precise strategies such as immune checkpoint blockade (ICB) (1) and chimeric antigen receptor T-cell (CAR-T) therapies (2), evidenced by melanoma 5-year survival rates reaching about 50% and leukemia remission rates exceeding 80%. However, the complex tumor microenvironment of solid cancers, characterized by hypoxia, acidosis, high interstitial pressure, and immunosuppressive conditions, continues to represent a significant obstacle to the effectiveness of existing therapies, primarily through mechanisms such as metabolic competition-induced T cell paralysis and physical exclusion of immune cells (3). Consistent with these progresses, treatments based on microorganisms in particular using attenuated *Salmonella* (*Salmonella enterica serovar Typhimurium*), have recently regained high interest due to their special biological characteristics (4).

Attenuated *Salmonella* is a facultative anaerobe that has a natural propensity to colonize tumors, invade them, and cross biological barriers that are generally impermeable to traditional chemotherapeutic agents (5, 6). Notably, recent developments in synthetic biology and artificial intelligence (AI) has established a new paradigm for engineered bacterial therapeutics. Leveraging these broader technological advances attenuated Salmonella is being transformed from a passive immune stimulant into a programmable therapeutic platform, featuring predictive circuit design, autonomous environmental sensing, and optimized payload delivery (7, 8). These synthetic bacteria could recognize tumor-specific signals, including hypoxia and necrotic products (mechanisms also described in *in vitro* tumor models) and process those signals using multilayered genetic circuits to perform precise therapeutic actions *in vivo* (9, 10). In response, they can deliver a range of therapeutic payloads, including cytotoxic agents (11), immunomodulatory factors (suan as CCL21 and IL-2) (12, 13), and tumor-associated neoantigens (14).

**Figure 1:**
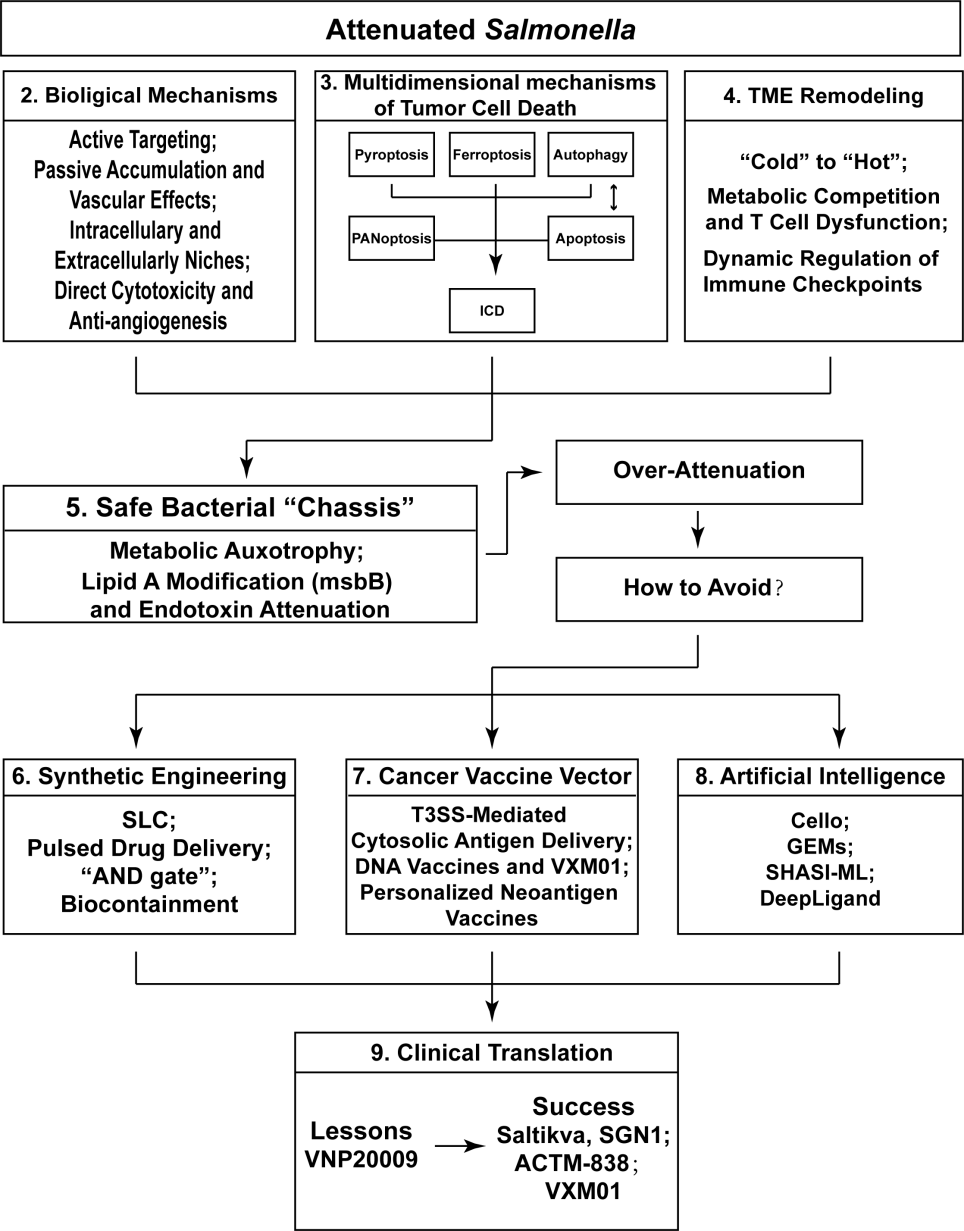
The specific flowchart and core content of this review.

This report aims to provide a comprehensive overview of the recent progress and emerging applications of attenuated *Salmonella* in cancer therapy. Particular emphasis is placed on detailed analyses of its tumor colonization mechanisms and its role in metabolic immune regulation within the TME, with special attention to the precise modulation of bacterial metabolism by host T cell immunity (15, 16). Approaches for constructing synthetic gene circuits, including platforms such as Cello (17), are introduced as the foundation for our discussion. These engineering approaches are further supported by evaluations of genome-scale metabolic models (GEMs) integrated with machine learning (ML) methods, such as SHASI-ML (18), which enable the simulation of metabolic fluxes to modulate nutrient competition and the precise identification of immunogenic neoantigens. In particular, these computational platforms have different prediction powers: GEMs enable metabolic flux analysis to influence the competition of nutrients (such as glucose and glutamine), whereas ML algorithms allow for the identification of neoantigens with high throughput and the evaluation of their immunogenicity with high precision. In addition, recent clinical trial findings from Saltikva (19) and ACTM-838 (NCT06336148: A Phase 1a/1b Study of ACTM-838 in Patients With Advanced Solid Tumors) are incorporated to inform perspectives on the future development of this field. The specific flowchart of this review is shown in **Figure 1**.

## 2 Biological mechanisms of attenuated *Salmonella*-mediated cancer therapy

Attenuated *Salmonella* is well-suited as a tumor-targeting vector due to its evolutionary adaptation to survive in harsh environments (5, 20). Despite being genetically weakened, these strains maintain strong tumor-targeting properties, achieving colonization levels in preclinical murine models that are 1,000 to 10,000 times higher than in normal tissues (4).

### 2.1 Active targeting mechanism: chemotaxis and receptor sensing

Attenuated *Salmonella* colonizes tumors not only through passive entrapment but also through active targeting by chemotaxis. Rapidly growing solid tumors with an aberrant vascular structure often contain large necrotic regions inside the tumor mass (21), which expel small nutrient molecules and other signaling molecules, including serine, aspartate, and ribose (9, 22). In addition, attenuated Salmonella have specific chemical receptors on their surface (Tar for aspartate and Trg for ribose) that enable them to sense these signals. They move up along concentration gradients through flagellar motion, which enables them to penetrate deeply into tumors and actively target hypoxic centers that are typically considered inaccessible to conventional chemotherapeutic agents (23). Despite being facultative anaerobes, attenuated *Salmonella* can grow under both aerobic and anaerobic conditions. In a normal oxygenated tissue, the exposed Salmonella are vulnerable to being neutralized by the host immune system (24); in contrast, the hypoxic, necrotic regions of tumors are characterized by compromised host immune surveillance and an abundance of nutrients, which provide a favorable habitat that enhances the colonization and proliferation of attenuated *Salmonella* in the TME (25).

### 2.2 Passive accumulation and vascular effects

In addition to active chemotaxis, the pathophysiological characteristics of the tumor vasculature also support bacterial enrichment through passive transport-related mechanisms and vascular effects. The enhanced permeability and retention effect is a critical factor in this context (26, 27). The disorganized architecture of tumor neovasculature, coupled with the presence of large fenestrations in the endothelium, facilitates the extravasation of macromolecules and particles from the blood circulation into the tumor interstitium (28). Furthermore, attenuated *Salmonella* can induce intravascular inflammation leading to vascular entrapment, hemorrhage, and ischemia (29). Bacterial-induced vascular damage, as shown experimentally with TNF-α-mediated hemorrhage and downregulation of proangiogenic factors including HIF-1α and VEGF, also contributes to tumor hypoxia and necrosis. This leads to a beneficial niche for anaerobic bacterial growth, and they form a positive feedback loop that reinforces itself continuously (30).

### 2.3 Colonization niches both intracellularly and extracellularly

The precise localization of attenuated *Salmonella* in the TME has remained a hot topic of study and is a critical aspect to elucidate the mechanism of therapy. Covered by effector proteins encoded on SPI-1 and SPI-2 of the *Salmonella* Pathogenicity Island (SPI) Type III Secretion System (T3SS) (31), attenuated *Salmonella* is a natural tumor cell invader that can form intracellular niches within tumor cells, a step essential for antigen presentation and immunogenic cell death (ICD) (32). Nevertheless, the assumption that this species favors strictly intracellular conditions has been recently challenged by studies based on the chick embryo chorioallantoic membrane (CAM) tumor model coupled to mathematical modeling, which suggest that most bacterial localization occurs through extracellular niches (33). The CAM tumor model is a valuable preclinical model for studying the tumor-colonizing process of Salmonella. It is a highly vascularized, partially immune-deficient extra-embryonic membrane that allows for rapid tumor xenografting and real-time observation of bacterial infection. Besides, Crull et al. (2011) (34) first confirmed that attenuated *Salmonella* forms biofilms within the *in vivo* TME. This process is regulated by key genes *csgD* and *adrA*, primarily serving as a defense mechanism against phagocytosis by host neutrophils. The formation of these biofilms explains why therapeutic *Salmonella* is consistently localized in extracellular niches within the tumor. Furthermore, recent imaging has identified extracellular bundles of bacteria within the TME (35), suggesting a complementary model where intracellular bacteria drive direct cytotoxicity while sustained high-density extracellular populations are vital for cytokine induction. Therefore, the therapeutic efficacy may be more reliant on a “dual-colonization” model involving efficient intracellular invasion and robust extracellular persistence.

### 2.4 Direct cytotoxicity and anti-angiogenesis

**Figure 2:**
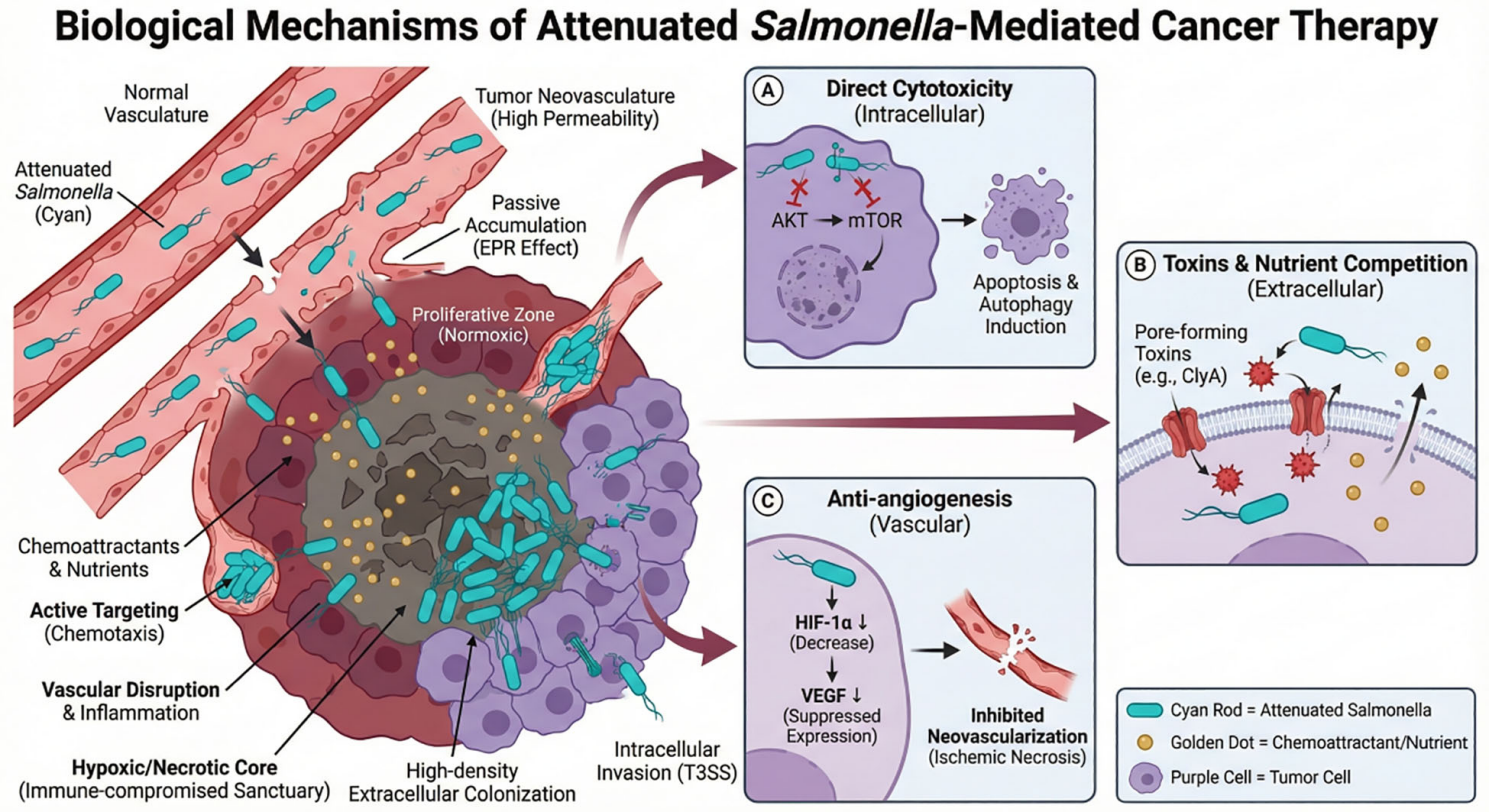
Biological mechanisms of attenuated *Salmonella*-mediated cancer therapy. This image is a demonstration of attenuated *Salmonella* target and colonization, indicating its systemic dissemination and route of penetration from the vasculature into the hypoxic/necrotic tumor center through the EPR effect (passive accumulation) and chemotaxis (actively targeting). Subsequently, they are able to produce antitumor effect by 3 major ways: **(A)** Inducing apoptosis and autophagy intracellularly through AKT/mTOR pathway inhibition; **(B)** Secreting pore forming toxins (such as ClyA) extracellularly that disrupt the integrity of tumor cell membranes and competing for nutrients; **(C)** Suppressing angiogenesis by down-regulation of HIF-1α and VEGF expression in ECs resulting in ischemic necrosis.

Upon successful colonization, attenuated Salmonella directly inhibits tumor cell growth by regulating cellular signaling pathways and secreting toxins. Studies reveal that infection with attenuated *Salmonella* significantly suppresses the intracellular AKT/mTOR signaling pathway, triggering metabolic reprogramming that directly activates autophagy and apoptosis, ultimately leading to cell death (36). In addition, as bacteria and tumor cells compete for the same nutrients during growth (28), *Salmonella* releases extracellular pore-forming toxins, such as ClyA (37) or other exotoxins that disrupt the integrity of tumor cell membranes. Simultaneously, it suppresses angiogenesis at the molecular level by lowering hypoxia-inducible factor-1α (HIF-1α) expression, and consequently reduces vascular endothelial growth factor (VEGF) expression. This deprives the tumor of neovascular supply and induces necrotic cell death via ischemia due to a lack of blood supply (38). The mechanism diagram of attenuated *Salmonella*-mediated cancer therapy is shown in **Figure 2**.

## 3 Multidimensional mechanisms of attenuated *Salmonella*-induced tumor cell death

After characterizing the mechanisms of tumor colonization and nutrient depletion, we then examine how these macroscopic interactions translate into specific death programs at the molecular level in tumor cells. Upon infecting tumor cells, attenuated *Salmonella* triggers multiple programmed cell death (PCD) pathways, including apoptosis (22), autophagy (36), and pyroptosis (39). These PCD pathways directly mediate tumor regression and are often strongly immunogenic (with features of ICD). This response triggers the release of damage-associated molecular patterns (DAMPs) into the system, which primes and activates antigen-specific anti-tumor immunity (40).

### 3.1 Pyroptosis: the epicenter of the inflammatory storm

Pyroptosis is a necrotic, pro-inflammatory type of caspase-dependent PCD with Gasdermin proteins as the executioner, promoting cell swelling and explosive cell lysis (41).

#### 3.1.1 Dual activation of canonical and non-canonical inflammasome pathways

Attenuated *Salmonella* can potently induce pyroptosis through two distinct mechanisms:

##### 3.1.1.1 The canonical pathway

Cytosolic translocation of attenuated *Salmonella* T3SS components (e.g., the rod protein PrgJ) and flagellin promotes their specific binding to NAIP receptors and subsequently to the NLRC4 inflammasome (42). This NLRC4-Caspase-1 interaction promotes NLRC4 inflammasome multimerization and activates Caspase-1. Activated Caspase-1 then cleaves Gasdermin D (GSDMD), releasing its N-terminal pore-forming region (GSDMD-N) to form membrane pores, and simultaneously processes pro-IL-1β and pro-IL-18 into their mature, active forms (41).

##### 3.1.1.2 The non-canonical pathway

Lipopolysaccharide (LPS) from the outer membrane of attenuated *Salmonella* is directly detected by cytosolic Caspase-4/5 in humans and Caspase-11 in mice. These activated inflammatory caspases cleave GSDMD like Caspase-1, initiating pyroptosis (43). In addition, this pathway amplifies inflammation through a positive feedback loop: caspase-dependent cleavage of the Pannexin-1 channel releases ATP, which activates the P2X7 receptor and subsequently stimulates the NLRP3 inflammasome (44).

#### 3.1.2 Gasdermin E: conversion of a molecular switch

Although GSDMD is often epigenetically silenced in many tumor cells, GSDME is commonly overexpressed. Traditional chemotherapeutic agents trigger apoptosis through activation of Caspase-3 (45). However, when GSDME is present, Caspase-3 preferentially cleaves GSDME to release the GSDME-N fragment, converting the otherwise “silent” apoptotic pathway into a robust pyroptotic pathway. Engineered attenuated *Salmonella* can induce Caspase-3 through the secretion of effector proteins such as SopF (46) or via metabolic stress-induced reactive oxygen species (ROS), selectively triggering pyroptosis in tumors with high GSDME expression (47). This strategy not only eliminates tumor cells but also releases HMGB1 and ATP, which strongly attract DCs and help overcome the immunosuppressive microenvironment (48).

### 3.2 Ferroptosis: lipid peroxidation driven by metabolic reprogramming

Ferroptosis is a regulated form of cell death that depends on iron and is characterized by the excessive accumulation of lipid peroxides. Studies conducted recently have demonstrated that attenuated *Salmonella*, particularly the engineered strain YB1, can remarkably induce ferroptosis in tumor cells (47).

#### 3.2.1 GPX4 degradation and the ROS burst

GPX4 is the primary intracellular defense enzyme against lipid peroxidation. However, its activity is strongly suppressed by attenuated *Salmonella* infection via a variety of convergent mechanisms. Proliferating bacteria metabolize a large amount of cysteine and glutathione, and the substrate required for GPX4 is depleted by rapidly growing bacteria (47). Moreover, the infection has been demonstrated in a systematic mechanistic way to promote the TAK1-HSC70 signaling axis to promote the lysosomal degradation of GPX4, which is a process related to the chaperone-mediated autophagy pathway (49). Inhibition of this enzyme is further enhanced by mitochondrial dysfunction induced by colonization with bacteria, which dissipates membrane potential and triggers ROS burst. The produced ROS then combine with intracellular labile iron through the Fenton reaction, further elevating lipid peroxidation and inducing ferroptosis (50).

#### 3.2.2 Ferritinophagy

Attenuated *Salmonella* infection simultaneously activates NCOA4-dependent ferritinophagy (51), a lysosomal degradation pathway that releases a large amount of labile iron. This “iron overload” pathway renders tumor cells highly sensitive to oxidative stress, resulting in membrane lipid peroxidation and cell death (30). Furthermore, co-administration of this approach with ferroptosis inducers, for instance Sulfasalazine, elicits a synergistic anti-cancer response (47).

### 3.3 Autophagy and apoptosis

#### 3.3.1 Autophagy: the balance between survival and death

Autophagy plays a dual role during attenuated *Salmonella* infection. Although host cells activate xenophagy to target and eliminate the bacteria (52), attenuated *Salmonella* can simultaneously manipulate the autophagic machinery to its advantage. This includes transiently promoting its intracellular survival or triggering autophagic cell death in host cells via effector proteins (e.g., SopB), which activate the AKT/mTOR pathway or physically bind to autophagy receptors such as p62 and NDP52 (53). Pharmacological inhibition of autophagy with drugs such as chloroquine (CQ) may increase the anti-tumor effects of attenuated *Salmonella* (54). This synergistic effect is caused by two mechanisms: the inhibitor prevents autolysosomal degradation, which diminishes the clearance of bacteria and results in an increase in the intracellular bacterial burden; and the inhibitor also inhibits the protective autophagic response of tumor cells to metabolic stress, thereby enhancing their susceptibility to bacterial cytotoxicity.

#### 3.3.2 Tipping the balance: the shift toward apoptosis

Autophagy is thought to have initially evolved as a homeostatic mechanism to eliminate intracellular bacteria and other pathogens to promote cell survival. However, bacterial-driven stress leads to the collapse of this defense, and a switch toward “autophagic cell death” is triggered (55). Within this pathway, the blockage of the AKT/mTOR pathway is a critical event. At an early stage, this inhibition induces autophagy; however, a long-term lack of survival signals at a later stage triggers mitochondrial/caspase-dependent apoptosis, which involves events such as Bax/Bak oligomerization (56, 57). From a therapeutic perspective, this presents a dual strategy: combining autophagy inhibitors, such as CQ, with attenuated *Salmonella* strains, such as A1-R, has been shown to enhance tumor elimination (54). This synergy demonstrates that autophagy can serve as a cytoprotective mechanism in response to stress induced by bacteria in certain situations, and its inhibition leads to the rapid induction of apoptosis. Conversely, other studies reveal that excessive autophagy can itself become the primary driver of cell death, highlighting that the effectiveness of these therapeutic strategies is influenced by the specific metabolic characteristics of the tumor (56).

#### 3.3.3 Apoptosis: the direct “suicide” command

Apart from autophagy-induced apoptosis, attenuated *Salmonella* strains with tumor-targeting characteristics can cooperatively induce cancer cell apoptosis through the intrinsic and extrinsic core pathways. In the intrinsic pathway, attenuated *Salmonella* infection generates oxidative stress, which upregulates the pro-apoptotic Bax protein, downregulates the anti-apoptotic Bcl-2 protein, disrupts mitochondrial integrity, enhances cytochrome *c* release, and triggers caspase-9/3 cascade to induce apoptosis (58). Simultaneously, through the extrinsic pathway, the infection induced an increase in the expression of death receptors targeted by TNF-α on tumor cell surface, thereby activating caspase-8 and the apoptotic machinery from the outside-in (59, 60). Attenuated *Salmonella* can also modulate intracellular signaling pathways by inhibiting the pro-survival AKT/mTOR pathway and reducing NF-κB activity to sensitize tumor cells to cell death and by activating the tumor suppressor p53 to enhance cell death signals (36). Taken together, through a three-pronged mechanism involving mitochondrial damage, immune-mediated cytotoxicity, and signaling pathway reprogramming, attenuated *Salmonella* potently triggers apoptosis in tumor cells. **Table 1** shows the comparison of direct apoptosis and autophagy-mediated apoptosis.

**Table 1:** Comparison of direct apoptosis vs. autophagy-mediated apoptosis in tumor cells by *Salmonella*.

Feature	Direct apoptosis	Autophagy-mediated apoptosis
Triggers	Bacterial toxins, mitochondrial damage, death receptor activation	Nutrient deprivation, metabolic stress, inhibition of the Akt/mTOR pathway
Key Protein	Caspase-3, Caspase-9, Cytochrome c	Beclin-1, LC3, Atg proteins
Process Description	Rapid execution of cell death	Initial survival attempt (autophagy) transitions to death upon failure
Role of *Salmonella*	Acts directly as the “executioner”	Acts as a “stressor,” forcing systemic cellular collapse

### 3.4 PANoptosis: the comprehensive collapse of death pathways

The recent concept, “PANoptosis,” refers to a distinct form of cell death in which pyroptosis, apoptosis, and necroptosis are activated simultaneously (61). Attenuated *Salmonella* infection promotes the formation of a PANoptosome complex containing Caspase-1, Caspase-8, RIPK1, and RIPK3. This integrated death machinery prevents tumor cells from evading cell death through mutations in a single pathway, such as Caspase-3 deficiency, highlighting a key advantage of bacterial therapy in overcoming tumor resistance (62).

### 3.5 Convergence on ICD

**Figure 3:**
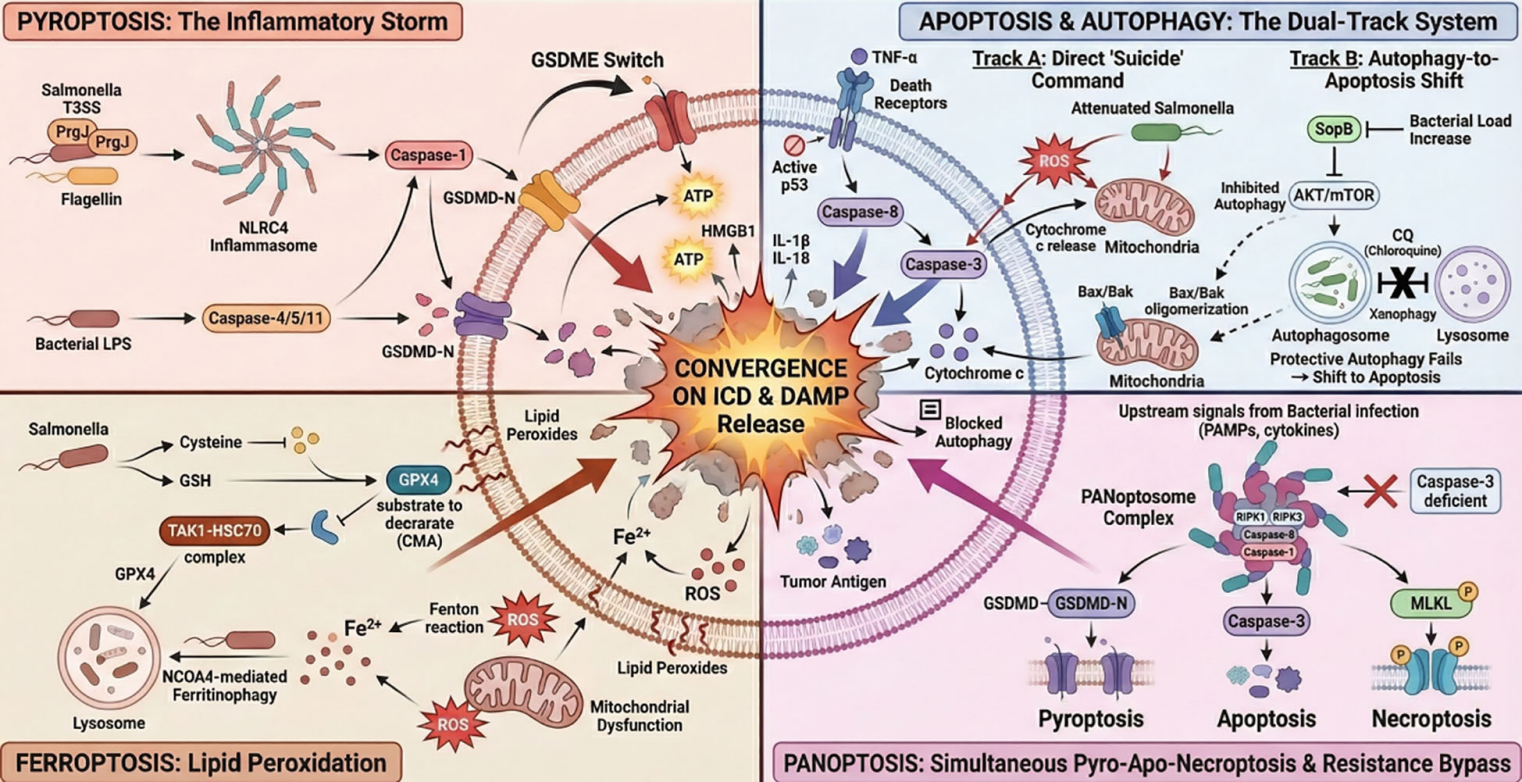
Multidimensional mechanisms of attenuated *Salmonella*-induced tumor cell death and ICD. *Salmonella* is known to activate the following four major PCD pathways, as illustrated in the diagram. (Top Left) Pyroptosis can be triggered through two inflammasome-dependent pathways: the canonical (NLRC4/Caspase-1) and non-canonical (Caspase-4/5/11) pathways, which result in GSDMD cleavage. Importantly, the GSDME switch can be activated by caspase-3 in pyroptosis (GSDME switch). (Bottom Left) Ferroptosis is induced by bacteria-mediated consumption of cysteine/GSH and FPC degradation of GPX4 through the TAK1-HSC70-mediated chaperone-mediated autophagy (CMA), as well as NCOA4-dependent ferritinophagy, which, in turn, enhances Fenton chemistry and lipid peroxidation. (Top Right) A two-track mechanism regulates the transition from autophagy to apoptosis. Direct bacterial stress (ROS, TNF-α) induces both intrinsic/extrinsic apoptosis, but therapeutic blockade (e.g., Chloroquine) or chronic stress can transform cytoprotective autophagy into apoptosis by inhibiting the AKT/mTOR pathway. (Bottom Right) PANoptosis incorporates molecules representing the different pathways (RIPK1/3, Caspase-1/8) to form the PANoptosome, and suppressing immune evasion. (Center) All of the pathways converge in affecting the stability of the membrane, which ultimately leads to the explosive release of DAMPs (ATP, HMGB1) and tumor antigens, a feature of such a robust ICD. In this diagram, the central region represents the intracellular space (cytosol), which is separated from the surrounding extracellular environment by the phospholipid bilayer (cell membrane).

Collectively, it is clear that none of the aforementioned PCD pathways function independently, they all intersect to provide a strong ICD response. Among these is pyroptosis, typically at the center of the inflammatory storm, in which bacterial T3SS machinery and LPS trigger canonical (NLRC4/Caspase-1) and non-canonical (Caspase-4/5/11) inflammasomes, respectively. This results in the formation of Gasdermin D-mediated pores and the violent release of biologically active IL-1β, IL-18, and DAMPs (41, 42). This immunogenicity is further amplified by ferroptosis, driven by bacterial consumption of cysteine and TAK1-HSC70-mediated GPX4 degradation, which results in lethal lipid peroxidation (47). Furthermore, the induction of PANoptosis via the PANoptosome complex prevents tumor escape mechanisms associated with single-pathway deficiencies (62). Even autophagy and apoptosis shape this landscape; pharmacological inhibition or prolonged stress leads to a shift in the balance towards apoptosis, which, under certain conditions, can be converted into highly immunogenic pyroptosis in GSDME-expressing tumors (48). Ultimately, these synergistic modes of death induce the systemic discharge of essential DAMPs (e.g., ATP, HMGB1) as well as tumor antigens. The massive release of immunostimulatory signals provides the immune system with “danger signals” to activate, forming the mechanistic basis for the extensive TME reprogramming elaborated in the next section. The multidimensional mechanisms of attenuated *Salmonella*-induced tumor cell death and microenvironment remodeling are shown in **Figure 3**.

## 4 Remodeling of the tumor microenvironment

The fundamental mechanism of attenuated *Salmonella* therapy involves transforming “cold” tumors to “hot” tumors (4). This transition is marked by extensive immune cell infiltration, robust cytokine production, and profound metabolic reprogramming.

### 4.1 From immunosuppression to immune activation

#### 4.1.1 Awakening of innate immunity

Attenuated *Salmonella*-associated PAMPs, such as lipopolysaccharide (LPS) and flagellin, act as potent agonists for TLR4 and TLR5, respectively, initiating an intense immune response. This stimulation induces a localized cytokine surge, characterized by high levels of TNF-α, IL-1β, IL-6, and IL-12 (63). Notably, TNF-α (29) shows antiangiogenic properties and can disintegrate tumor vasculature as well as increase vascular permeability, which subsequently promotes immune cell infiltration. Attenuated *Salmonella* also reprograms macrophage phenotype from pro-tumor M2 subtype to anti-tumor M1 subtype, leading to the production of nitric oxide (NO) and reactive oxygen species (ROS) that directly lyse tumor cells (11, 64). Additionally, intracellular invasion of tumor cells with attenuated *Salmonella* leads to NLRC4 and NLRP3 inflammasome formation and caspase-1 activation, culminating in mature IL-1β and IL-18 secretion, which triggers tumor cell pyroptosis (further details are provided in section 4.1), an “explosive” and highly immunogenic form of cell death (16, 30).

#### 4.1.2 Bridging to adaptive immunity and immunological memory

The activation of innate immunity favors the development of adaptive immunity. Following their activation in the inflammatory environment, dendritic cells (DCs) endocytose both tumor and bacterial antigens and migrate to lymph nodes to prime CD4^+^ and CD8^+^ T cells (30). In addition to acting as an inherent adjuvant, attenuated *Salmonella* promotes antigen cross-presentation by killing tumor cells, leading to the release of neoantigens and subsequently inducing strong cytotoxic T lymphocyte (CTL) responses against tumor-specific antigens (65). This widespread T cell activation underlies the abscopal effect, where local administration of attenuated *Salmonella* can trigger regression of distant, untreated tumors (66).

### 4.2 Key findings: metabolic competition and T cell dysfunction

Although attenuated Salmonella strains are able to recruit T cells actively, clinical and preclinical data often report exhaustion or hyporesponsiveness of T cells in tumors of treated patients (67). High-dimensional analysis revealed a central underlying phenomenon: metabolic competition (68). This is a breakthrough result in the understanding of bacteria-host interaction in the tumor microenvironment.

#### 4.2.1 The asparagine-c-Myc axis

A report in *EMBO Molecular Medicine* on a first-class study revealed a fundamental trade-off for attenuated *Salmonella* as a cancer therapeutic, by showing that tumor growth inhibition through metabolic competition with cancer cells could be antagonized by optimal T cell-mediated immunity (69). L-bacterial asparaginase (AnsB) secretion depleted asparagine in the TME and mechanistic studies in murine CRC models demonstrated. This depletion reduced proliferation of tumor cells addicted to the amino acid, but also hindered the metabolic rewiring of activated T cells. Quantitative analysis indicated that removal of asparagine from the metabolic micro-environment destabilized c-Myc metabolism, leading to cell cycle arrest in T cells and significantly reduced production of effector cytokines such as IFN-γ (70). To be more precise, c-Myc destabilization, a key metabolic regulator that reactivates T cells from resting state to a highly proliferative effector with enhanced glycolysis and oxidative phosphorylation, is induced by asparagine depletion. Consequently, c-Myc–deficient T cells, lacking c-Myc, fail to sustain metabolic reprogramming in response to signals through the T-cell receptor (TCR), arrest in the cell cycle, and produced fewer cytokines including IFN-γ. This state, which is distinct from classical immune checkpoint exhaustion, has recently been labeled as “metabolic paralysis.”

This state of “metabolic paralysis” represents a profound biological “yin-yang” dilemma (71): the necessity to starve the tumor while preserving the metabolic fitness of immune cells. Recent findings by Qiao et al. (2026) (72) further underscore that asparaginase expression—a feature shared by other oncolytic vectors such as engineered E. coli Nissle 1917—can profoundly impair T-cell responses. This suggests that the Asparagine-c-Myc axis is a universal consideration in bacterial immunotherapy, necessitating advanced engineering strategies to balance nutrient competition with effective anti-tumor immunity.

#### 4.2.2 Engineering trade-offs

In one study, asparagine was repleted in the TME through the deletion of the *ansB* gene in attenuated *Salmonella* (Δ*ansB*). The results showed that although this measure restored metabolic and effector function of T cells, it also provided nutrients to promote tumor cell growth. However, in certain colorectal cancer models, the Δ*ansB* mutant successfully controlled tumor growth by a mechanism involving re-established immune surveillance (70). Our observations highlight the importance of a finely tuned balance between suppressing tumor metabolism and supporting immune cell metabolism while designing strains.

### 4.3 Dynamic regulation of immune checkpoints

Attenuated *Salmonella* has a complex, reciprocal regulation of immune checkpoints, including PD-L1. Infection with attenuated *Salmonella* downregulates PD-L1 expression on tumor cells by inhibiting the intracellular AKT/mTOR/p70S6K signaling pathway, thereby enabling tumor suppression by CD8^+^ T cells (73). In contrast, the robust inflammatory response induced by the infection, with massive secretion of IFN-γ, frequently leads to compensatory upregulation of PD-L1 by immune cells (DCs and B cells) and certain tumor cells, as a negative feedback process to avoid an exaggerated immunopathology (74, 75). Although this immune-driven upregulation of checkpoint pathways may partially limit the anti-tumor effects of attenuated *Salmonella* monotherapy, it provides a compelling rationale for combining it with anti-PD-1/PD-L1 antibodies. In such combinations, attenuated *Salmonella* functions as a T cell recruiter to convert an immunologically “cold” tumor into “hot,” while checkpoint inhibitors enhance the survival and activity of the infiltrating effector cells (74, 76).

## 5 Attenuation strategies: engineering a safe bacterial “chassis”

Although the profound TME remodeling and immune activation described above will be responsible for therapeutic efficacy, it could also lead to systemic toxicity. Therefore, balancing this strong immunogenic property with safety by suitable attenuation is a prerequisite for clinical use. The advancement of attenuated *Salmonella*-based therapies into clinical development depends upon the resolution of the safety issue. Because wild-type *Salmonella* Typhimurium is pathogenic (causing severe gastroenteritis and sepsis that may be fatal), strategies for attenuation must remove virulence while maintaining, or even improving, the bacterium’s tumor colonization and immune activation capabilities. Achieving this delicate balance, often referred to as the “immunogenicity-safety trade-off,” remains a central challenge (77, 78).

### 5.1 Metabolic auxotrophy as an attenuation strategy

Metabolic auxotrophy is a classic attenuation approach in which one or more critical genes in vital metabolite biosynthetic pathways are deleted, thereby causing the bacterial cell to rely on an exogenous supply of the targeted metabolite. This design is primarily based on the metabolism of metabolites, which usually have high concentrations in the TME and low concentrations in healthy tissues and blood. For example, the deletion of purI blocks purine biosynthesis (the precursor of VNP20009); furthermore, because free purines are virtually absent in normal tissues but are abundant in the necrotic tumor debris, purI mutants cannot proliferate systemically but can grow in the center of tumors (79). Preclinical biodistribution studies have shown that this strategy for metabolic sequestration leads to colonization ratios of tumor to normal tissue in excess of 1,000:1, creating a substantial window for therapeutic intervention (4). The aroA mutation also generates a highly attenuated phenotype that is extensively used in vaccine vectors such as strain SL7207 (refer to allele nomenclature above) (80). Similarly, the new generation of therapeutic candidates, such as ACTM-838, is based on auxotrophic approaches (targeting purine/adenosine dependency) and utilizes a two-pronged strategy (adenosine abundance and immunosuppressive TME) to limit bacterial proliferation in normal tissues whilst promoting selective tumor site enrichment (81).

### 5.2 Lipid a modification (*msbB*) and endotoxin attenuation

Lipid A, a toxic component of LPS, initiates septic shock through TLR4 signaling. The *msbB* gene product, myristoyltransferase, is required for the final step of lipid A acylation, and its absence leads to the production of penta-acylated lipid A instead of hexa-acylated lipid A (82). This modification substantially decreases bacterial toxicity to less than 1/10,000th, greatly diminishing the risk of cytokine storm and septic shock development, and enabling the use of a higher dose of attenuated bacteria in patients. However, this attenuation approach has a drawback: decreased engagement of TLR4 implies elicitation of fewer potent antitumor cytokines such as TNF-α (83). This “over-attenuation” is often cited as a reason for the clinical failure of VNP20009, where the immune system cleared the bacteria before they could effectively colonize the tumor environment (19).

### 5.3 The cautionary tale of over-attenuation

However, lipid A modification through deletion of msbB reasonably reduces toxicity (82), at the expense of a substantial “immunoreactivity- safety trade off.” The consequent decrease in TLR4 signaling attenuates the generation of pro-inflammatory cytokines, including TNF-α, which are necessary for vascular disruption and the recruitment of immune cells (79). This phenomenon of “over-attenuation” is exemplified by the strain VNP20009 (84). Although being engineered for safety, this bacterium was rapidly eliminated by the host immune system and did not attain a colonization density enough to produce a therapeutic effect (19). Moreover, a clear therapeutic window was not apparent as dose-limiting toxicities inhibited efficient colonization, underlining the difficulties inherent to the static gene deletion approach. This is an example of a setback illustrating the need for dynamic control strategies, such as the Synchronized Lysis Circuit (SLC) decoupling bacterial colonization and immunogenic lysis, to separate toxicity and immunogenicity rather than wholly eliminating the bacterium’s immune stimulating potential (85). The comparison of key antitumor attenuated *Salmonella* strains is shown in **Table 2**.

**Table 2:** Comparison of key antitumor attenuated *Salmonella* strains.

Strain name	Key genotype	Attenuation & targeting mechanism	Core status & characteristics
VNP20009	Δ*msbB*, Δ*purI*	Reduced LPS toxicity and Tumor-specific purine dependency	FDA approval for Phase I trials. Excellent safety profile, but demonstrated poor colonization and insufficient efficacy in humans (76).
A1-R	leu^-^/arg^-^	Dual leucine/arginine auxotrophy	Exhibits an extremely high tumor-targeting ratio (>1:10,000) while retaining virulence factors; effective against metastasis (63).
ΔppGpp	Δ*relA*, Δ*spoT*	Impaired intracellular survival and Potent inflammasome activation	Rapidly cleared in normal tissues due to stress intolerance, but induces a strong IL-1β cytokine storm within tumors (16).
SL7207	Δ*aroA*	Aromatic amino acid metabolic defect	Shows restricted *in vivo* growth; widely used as a carrier for the oral delivery of DNA vaccines (80).
YB1	Δ*asd*, *P_pepT_-asd*	Hypoxia promoter switch and obligate anaerobic conversion	Engineered via synthetic gene circuits to survive strictly in hypoxic zones, theoretically ensuring absolute safety (127).
Ty21a	*galE* mutation	Galactose metabolism defect (autolysis)	Licensed typhoid vaccine strain with the most comprehensive safety data for human use; adapted for clinical cancer trials (99, 100).

## 6 Synthetic biology-driven functional enhancement and intelligent delivery

With VNP20009 as an example of the limitations of static attenuation strategies, the field has moved towards dynamic control. Synthetic biology allows us now to transform these bacterial “chassis” into intelligent vectors that can sense and react to the TME.

### 6.1 Synchronized lysis circuit and pulsed drug delivery

Since bacterial colonization alone is frequently insufficient to eradicate tumors, synthetic biology has transformed attenuated *Salmonella* into an “intelligent vector,” engineered with autonomous density-sensing circuits to execute programmable lysis and maximize therapeutic payload delivery. To improve safety by controlling bacterial overgrowth and enhancing the delivery of therapeutic macromolecules, a biorobotic system called the Synchronized Lysis Circuit (SLC) was developed (10). SLC leverages quorum sensing, where bacteria are engineered to continuously produce signaling molecules, such as AHL. Once the bacterial population reaches a specific density, the accumulated AHL triggers the expression of a lysis gene, such as a bacteriophage lysis protein. This causes the synchronized lysis of more than 90% of bacterial cells, releasing intracellular molecules in a pulsed manner. A small fraction of bacteria survive, serving as a “seed” to repopulate and restart the cycle (86). A state-of-the-art study published in 2025 combined the SLC with the immunomodulatory LIGHT (87). The engineered bacteria were subject to periodic lysis inside tumors, releasing LIGHT, which bound to host HVEM receptors and promoted the development of mature tertiary lymphoid structures (mTLSs). Quantitatively, this strategy increased the proportion of activated CD69+ CD8+ T cells within the tumor from 3.5% to 23.1%, leading to complete tumor growth inhibition and significantly prolonged survival in colorectal cancer models. Functioning as immunological “outposts,” these mTLSs efficiently recruit and activate T cells and NK cells, overcoming the limitations of conventional immunotherapies, which often show limited efficacy against tumors.

### 6.2 Surface display and *in situ* synthesis

Using *Salmonella* surface display systems, such as the Lpp-OmpA fusion protein, tumor-specific antibodies (e.g., anti-CEA single-chain variable fragments, scFv) can be presented on the bacterial surface, enhancing targeting specificity (88). Furthermore, these engineered bacteria can be programmed to produce cytotoxic proteins like ClyA (89), cytokines such as IL-2, IL-18 (90), or prodrug-converting enzymes (e.g., cytosine deaminase (91)). This strategy allows for localized, high-concentration therapeutic delivery, minimizing systemic toxicity.

### 6.3 Logic gates and biosensors

**Figure 4:**
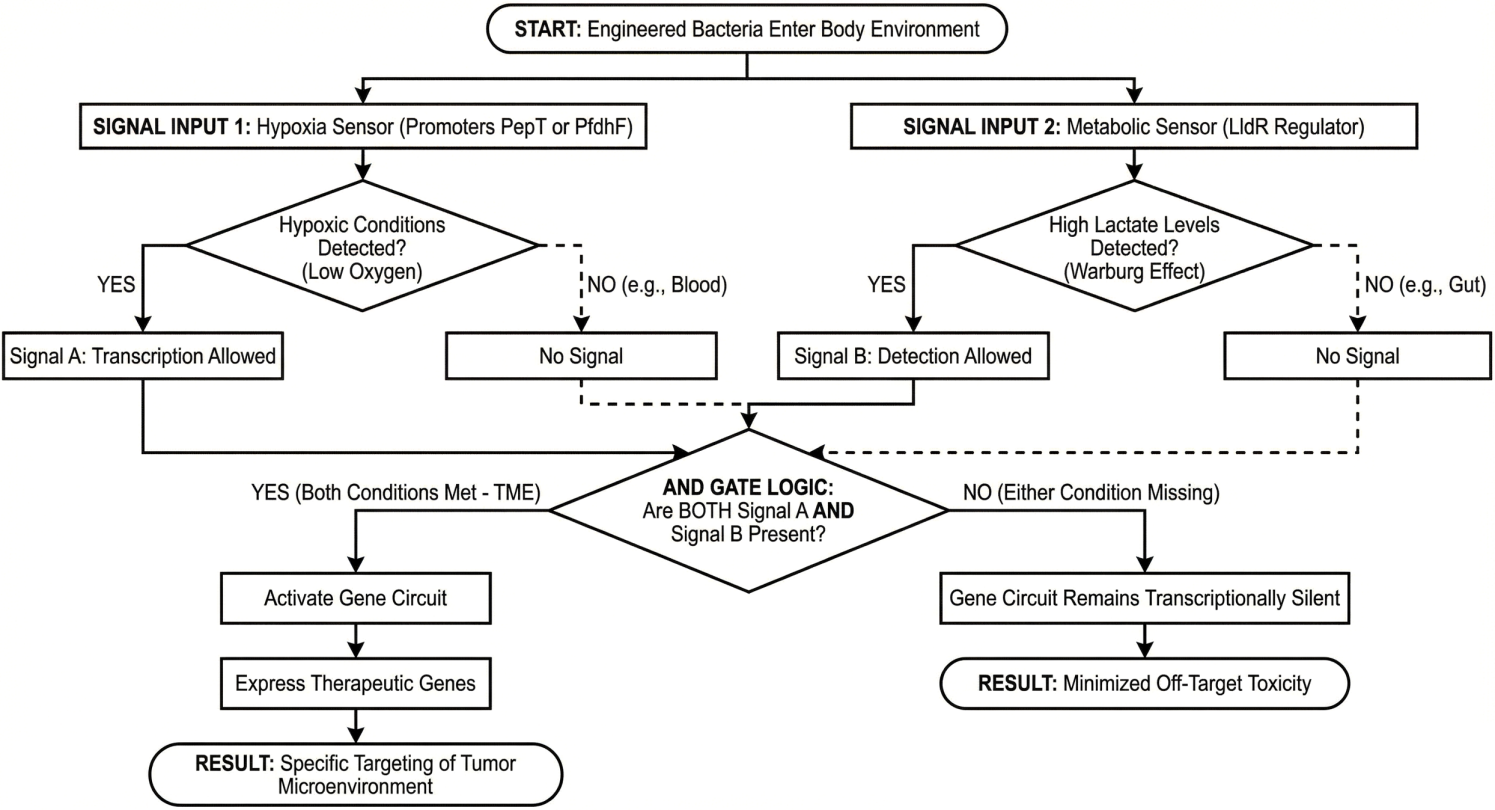
Schematic representation of the logic gate-based biosensor system for tumor targeting. For high specificity, a Boolean “AND” gate system, which combines two environmental signals, hypoxia and high lactate, has been implemented. The circuit integrates hypoxia-sensing promoters *PepT* or *PfdhF* and the metabolic sensor for lactate accumulation linked to Warburg metabolism, *LldR* regulator. The therapeutic gene circuit is triggered only by both signals at the same time, a TME hallmark. In normal tissues (e.g., gut or blood) where these conditions do not converge, the bacteria stay transcriptionally silent to avoid off-target toxicity.

To enhance targeting precision, “AND Gate” systems based on Boolean logic have been engineered (92). These systems rely on detecting multiple signals to activate therapeutic gene expression, a central design principle that underpins the tumor-specific functionality of the entire platform. In particular, this approach integrates hypoxia-responsive promoters, such as *PepT* or *PfdhF*, which are active only under low-oxygen conditions, with metabolic sensors like the LldR regulator that detect the elevated lactate levels characteristic of Warburg metabolism (92). Consequently, the genetic circuit is activated only in regions exhibiting both low oxygen and high lactate, hallmarks of the TME. This design keeps the bacteria transcriptionally inactive in the gut, which is hypoxic but metabolically distinct, and in the oxygen-rich bloodstream. *In vivo* characterization has empirically confirmed this spatial restriction, showing that the “AND gate” circuit tightly restricts expression of the therapeutic gene to the TME and remains off in healthy organs, leading to a dramatic decrease in off-target toxicity. The flow chart of the Logic Gates is shown in **Figure 4**.

### 6.4 Biocontainment and safety switches

Next-generation strains incorporate multiple biocontainment strategies to minimize the risk of environmental escape and genetic contamination. These advances, such as CRISPR-based self-destruction systems (93) and synthetic auxotrophies dependent on non-standard amino acids (94), ensuring both immediate and prolonged bacterial lethality outside the host environment. In addition, to enhance safety in clinical applications, “Kill Switches” responsive to specific antibiotics or metabolites have been engineered, enabling rapid and controlled elimination of the bacteria in the event of severe adverse reactions (95).

## 7 Attenuated *Salmonella* as a cancer vaccine vector

In addition to being a direct therapeutic agent or drug delivery vectors, the intracellular lifestyle of attenuated *Salmonella* makes them naturally ideal as a vector for cancer vaccines, especially for the cytosolic delivery of tumor antigens.

### 7.1 T3SS-mediated cytosolic antigen delivery

Attenuated *Salmonella* has the native capability to invade Antigen-Presenting Cells (APCs), making it a promising platform for the development of cancer vaccine vectors. To overcome the limitation of conventional protein vaccines, which primarily engage the MHC-II pathway and fail to elicit strong CD8+ T-cell (CTL) responses, we used the SPI-1 T3SS of attenuated *Salmonella* to deliver antigens directly into the cytosol (96). This method is based on the T3SS effectors (e.g., SopE) fusion with tumor-associated antigens (TAAs), so the SopE-TAA fusion can be directly “injected” into the APC cytosol (97). Once inside the cytosol, these antigens are processed by the proteasome and presented to the MHC class I pathway, resulting in strong CD8+ CTL responses. For example, the SopE-NY-ESO-1 fusion protein vaccine induced significant tumor regressions and promoted epitope spreading in a melanoma model (98).

### 7.2 DNA vaccines and VXM01

The DNA vaccine approach (Bactofection) uses attenuated *Salmonella* as a vector to transfer eukaryotic expression plasmids into the nucleus of APCs. VXM01, an oral T cell vaccine against vascular endothelial growth factor receptor 2 (VEGFR2), contains a plasmid encoding VEGFR2 and is based on the concept that this receptor is abundantly expressed on tumor vascular endothelial cells (99). Following oral intake, the bacteria are phagocytosed by M cells in Peyer’s patches, transfecting APCs. The results of clinical data from a Phase I/II study in patients with glioblastoma (GBM) indicated that VXM01 in combination with Avelumab (anti-PD-L1 antibodies) was well tolerated; a subset of patients showed an increased CD8+/Treg ratio, and the median overall survival (OS) was 11.1 months, indicating the capacity to mitigate immune tolerance and attack tumor vasculature (100).

### 7.3 Personalized neoantigen vaccines

Advances in sequencing technologies have spurred interest in vaccines targeting patient-specific mutated antigens (neoantigens), enabling a personalized immunotherapy approach (101). The attenuated *Salmonella* vectors are better suited to the rapid generation of personalized neoantigen vaccines because of their short construction cycle (a few weeks to generate engineered strains) (14). Advanced algorithms like DeepLigand allow for reliable neoantigen epitope prediction by taking relevant factors into account, such as the HLA binding affinity or the TCR recognition probability. The integration of these refined candidates into an attenuated *Salmonella*-based delivery platform has demonstrated the generation of personalized immunotherapy with strong tumor regression in animal models (102, 103).

## 8 Application of artificial intelligence in engineered bacteria design

### 8.1 Automated circuit design and component optimization

Traditional genetic circuit design for synthetic bacteria is typically based on inefficient trial-and-error approaches; however, the design of such circuits is being revolutionized by AI that facilitates predictable rational design. Researchers can now describe biological logic functions by using high-level programming languages reminiscent of Verilog, with automated tools such as Cello (17). Experimentally, Cello has been utilized to construct complex Boolean logic gates in gram-negative bacteria, improving the first-try experimental success rate of multi-gene circuits to an unprecedented 75% while effectively minimizing toxic signal crosstalk (17). AI-based algorithms compute the best combinations amongst all the standard components available to develop an optimal DNA device, considering that none of the sequences must be similar to any other sequence to avoid crosstalk; these algorithms also calculate penalties to avoid signal crosstalk, facilitating the automatic design of functional DNA sequences. Deep learning algorithms, including convolutional neural networks and generative adversarial networks, can predict and generate synthetic promoters (104) or ribosome binding sites (8) with given dynamic ranges for expression tuning of toxic proteins; they can also be utilized for the extensive analysis of genomic datasets. For instance, machine learning algorithms and biophysical models (such as the RBS Calculator) have been experimentally applied to optimize hypoxia-driven circuits in vectors like Salmonella, precisely tuning the release of therapeutic payloads only within the tumor hypoxic core and preventing premature systemic leakage (105, 106). Additionally, for systems with finely tuned parameters, such as SLC, the algorithm performs rapid multi-dimensional searches for optimal parameters that could enable the identification of bacterial lysis cycles that match tumor growth rates, without extensive wet lab experiments (10, 107). However, to ensure translational rigor, it is crucial to delineate these experimental milestones from future clinical realities. While AI-guided approaches have demonstrated remarkable precision *in vitro* and *in silico*, their robust and predictable performance within the highly dynamic *in vivo* human tumor microenvironment remains largely conceptual and requires extensive future clinical validation.

### 8.2 Genome-scale metabolic models and metabolic reprogramming

Combining GEMs with constraint-based modeling and flux balance analysis is a robust method to achieve global metabolic engineering of bacteria. Currently, high-quality attenuated *Salmonella* models iMA945 (108) and iRR1083 (109) have been developed for bacterial metabolic fluxes across varying microenvironments. By modeling gene deletion and predicting growth, this method can forecast the impact of the loss of individual metabolic routes on growth before experiments and help identify candidates of attenuation (*aroA* and *thyA*) to ensure that the engineered bacteria are safe in normal tissues but remain active in the TME (110). Recent studies have used these models to investigate the metabolic competition between attenuated *Salmonella* and cancer and immune cells. These studies explore mechanisms regarding how bacteria compete for essential nutrients (e.g., glucose and glutamine) (68), and inform the engineering of strains that robustly “starve” the tumor cells while sustaining the metabolism of antitumor T cells to counter immune suppression caused by bacterial nutrient scavenging (111).

### 8.3 Evolution of intelligent biosensors

In intelligent sensing, directed evolution approaches integrating microfluidics and ML are accelerating the development of high-quality biosensors. Guided by AI-based algorithms (112), the ultra-high-throughput screening of sensor variants from the vast mutation libraries with high sensitivity and specificity for trace tumor markers is documented, including specific proteases or low pH conditions (92).

### 8.4 AI-driven neoantigen prediction and vaccine design

Attenuated *Salmonella* is a good candidate vector for cancer vaccines, and AI is critical to identify neoantigens that can generate strong T cell responses. To select the optimum antigen for bacterial vectors, ML schemes based on Extreme Gradient Boosting (XGBoost) (e.g., SHASI-ML) have been proposed to combine global protein characteristics with sequence-based features (113). These methods achieve high precision and specificity in predicting bacterial immunogenic proteins and have been applied to screen hundreds of potential novel immunogens within the *Salmonella* proteome. For broader neoantigen prediction, deep models like DeepLigand use Natural Language Processing (NLP) techniques, treating peptide sequences as a language to capture the contextual relationships between amino acids (114). By predicting HLA binding affinity, antigen processing efficiency, and TCR recognition likelihood, these approaches enable the design of personalized “*Salmonella*-neoantigen” constructs tailored to an individual patient’s genomic profile. Experimentally, when such AI-selected neoantigens are engineered into attenuated bacterial vectors, they have successfully elicited robust, antigen-specific CD8+ T cell responses and significantly reduced tumor burden in preclinical murine models. Nevertheless, while platforms like SHASI-ML and DeepLigand have demonstrated high predictive accuracy *in silico* and in these preclinical settings, their direct translation into personalized clinical bacterial therapeutics is still in its infancy. Extensive human trials are required to validate whether these algorithmically predicted neoantigens can consistently overcome immune tolerance and trigger effective anti-tumor responses in patients.

## 9 Clinical translation: challenges and breakthroughs

While AI and synthetic biology chart the future of strain engineering, the ultimate validation of these concepts lies in human trials. We now review the lessons learned from past failures and the promise of recent clinical successes.

### 9.1 Lessons from VNP20009

VNP20009 (S. Typhimurium Δ *msbB* Δ *purI*) is a landmark strain in bacterial therapy history. However, despite outstanding preclinical performance, its Phase I clinical trial in metastatic melanoma and renal cell carcinoma was met with disheartening results (16). The clinical failure of VNP20009 was multifactorial, driven by severe compounding translational challenges including rapid immune clearance, dose-limiting systemic toxicity, and remarkably poor tumor colonization. Specifically, there was no apparent therapeutic window: the Maximum Tolerated Dose (MTD) was strictly capped at 3*10^8^ CFU/m², beyond which patients experienced severe dose-limiting toxicities (DLTs) at 1*10^9^ CFU/m², even with the msbB deletion, whereas lower, nontoxic doses were rapidly eliminated by the host defense system before effective tumor colonization could occur. Consequently, intratumoral colonization was extremely rare, detected in only 12% (3 out of 25) of patients at the highest, near-toxic doses. Furthermore, the colonization levels in patient biopsies were reported to be far below the threshold required for therapeutic activity. The average density observed was only 3*10^4^ CFU/g, which is several orders of magnitude below the critical level (~ 1*10^9^ CFU/g (5)) needed to induce tumor regression, resulting in a 0% objective tumor regression rate.

Adding to these physiological barriers, whole-genome sequencing later revealed a major reason for this failure: VNP20009 contained unintentional spontaneous mutations in the *rpoS* gene, encoding the stress-response sigma factor, and a silent mutation in *suhB*, reducing its metabolic fitness as well as its ability to colonize hepatocytes (79). Consequently, the failure of VNP20009 illustrates the limitations of “hard-wired” static gene deletions for bacterial attenuation and signals the need for a new generation of therapeutics governed by dynamic gene circuits and synthetic biology (72).

### 9.2 The success of Saltikva (*Salmonella*-IL2)

In sharp contrast to the setbacks with VNP20009, Saltikva (an attenuated *Salmonella* strain engineered to express human interleukin-2) has shown positive clinical breakthroughs in patients with pancreatic cancer (13). In this Phase II clinical trial (NCT04589234) of Saltikva in combination with FOLFIRINOX in patients with metastatic pancreatic cancer, the combination showed more effectiveness compared to historical data: the data revealed a median progression-free survival of 15 months for the combination treatment, which was considerably better than the historical benchmark (5.8 months) for FOLFIRINOX alone (115, 116), and the median OS (mOS) increased from the historical baseline of 11.5 to 20.3 months. Additionally, the study established the safety of oral delivery and the engendering of systemic immune activation through flow cytometry, demonstrating a dramatic increase in peripheral NK cells and NK-T cells. These results represent a significant milestone and demonstrate that attenuated *Salmonella*-based immunotherapy may induce systemic immune remodeling with substantial clinical benefits even in refractory solid tumors.

### 9.3 Other emerging clinical trials

As mentioned above, several emerging clinical trials are in progress, which testify to the variety of the pipeline for attenuated *Salmonella*-based therapeutics. ACTM-838 (Actym Therapeutics) is designed to co-deliver IL-15 with a STING agonist for the dual activation of innate and adaptive immunity (81); its safety is currently being assessed in a Phase I study (NCT06336148) in patients with advanced solid tumors. For relapsed refractory malignancies, VXM01, an oral T cell vaccine against VEGFR2, has shown promising results. In a Phase IIa study (NCT03750071) for recurring GBM, VXM01 in combination with the PD-L1 inhibitor avelumab achieved an overall response rate of 12% and a mOS of 11.1 months, which is a major positive indication for a disorder historically associated with dismal prognosis (117). Additionally, a multicenter clinical trial for advanced solid tumors is currently in progress and involves the *Salmonella* SGN1 strain. Most tumor cells are unable to synthesize methionine autonomously (Hoffmann effect) (118); by utilizing this vulnerability of tumor cells, the SGN1 strain, which expresses L-methioninase, continuously depletes methionine within tumors and blocks tumor growth. The strain has proven to have a manageable safety profile, mainly consisting of transient grade 1/2 pyrexia. In terms of effectiveness, initial findings suggest therapeutic advantage as demonstrated by quantifiable tumor reduction (Partial Response) and prolonged Stable Disease (SD) in cases of treatment-resistant solid tumors (NCT05038150 and NCT05103345).

## 10 Challenges and future perspectives

Despite promising prospects, the widespread clinical application of attenuated *Salmonella*-based therapy must surmount several critical technical and regulatory hurdles.

### 10.1 Genetic stability and evolutionary escape

Plasmid loss is common for engineered bacteria in the absence of *in vivo* selection with antibiotics; retention rates are known to be below 10% after approximately 50 generations. While balanced lethal systems, such as *asd* gene complementation, are widely employed to maintain plasmid stability at a high rate of exceeding 95%, they do not completely impede “evolutionary escape.” In this process, bacteria acquire mutations that shut down the expression of metabolically costly or toxic proteins to restore their fitness. Addressing this instability remains a major challenge in synthetic biology, and it is an area where AI-assisted design is expected to have the greatest impact (119).

Although some engineered bacteria utilizing genomic integration have entered clinical trials, Cubillos-Ruiz et al. explicitly pointed out in *Nature Reviews Drug Discovery review* (120) that maintaining the long-term, stable expression of these complex genetic circuits remains a massive technological chasm. This is particularly evident in the highly complex, exceptionally heterogeneous human solid tumor microenvironment, which completely lacks antibiotic selection pressure. Likewise, translating other advanced containment strategies—such as metabolic addiction (auxotrophy) circuits (121)—into such demanding *in vivo* settings safely and robustly continues to pose a significant challenge for current clinical translation.

### 10.2 Immune clearance and toxicity balance

Following systemic administration, the host immune system acts as a formidable biological barrier. The reticuloendothelial system (RES)—primarily the liver and spleen—typically clears >90–99% of the injected bacteria from the bloodstream within hours, often leaving less than 0.1% of the initial dose to successfully reach the tumor (122). Mechanistically, the complement cascade rapidly opsonizes or directly lyses the circulating bacteria, while neutrophils and tissue-resident macrophages efficiently engulf and destroy them through phagocytosis and respiratory bursts (123). Furthermore, pre-existing anti-*Salmonella* antibodies, which are highly prevalent in the human population, significantly accelerate this antibody-dependent clearance before effective tumor colonization can occur. This massive systemic bottleneck represents a major translational challenge. To overcome this, present studies are directed towards introducing polymer coatings (e.g., PEGylation) or cell membrane coating technologies (using erythrocyte or cancer cell membranes) to confer immune evasion  (124). In addition, a TNF-α surge or a “cytokine storm (29),” can be induced by high doses of attenuated strains. Crucially, the relationship between bacterial attenuation and immunogenicity is not always a traditional linear trade-off. Certain attenuated strains can exhibit unexpected immunological effects; for instance, the *aroA* mutant has been shown to paradoxically induce higher TNF-α production than its unattenuated counterparts (125). This nuance further emphasizing the importance of dose-control strategies that are precise, predictive, and clinically translatable. To this end, new synthetic methodologies utilize quorum-sensing circuits to impose a self-limitation on bacterial population densities (10). In a similar vein, PK modeling is applied to define the best dosing regimens for distancing therapeutic efficacy from systemic toxicity.

### 10.3 Environmental shedding and biosafety

Attenuated *Salmonella* are live bacteria and can be released into the tumor microenvironment through patient excretion, with the potential for transmission. Therefore, the development of stringent genetic “kill switches” or strict auxotrophic containment systems is essential for regulatory approval (126).

## 11 Conclusion

Attenuated *Salmonella* tumor therapy is now emerging from the first generation of statically derived attenuated strains to a precision medicine approach enabled by synthetic biology and AI. Initial clinical failures, like that seen with VNP20009, have enlightened: a bacterial therapy for maximal efficacy should not be based on the removal of overall virulence, but on fine-tuned spatiotemporal control, provided by tools such as synthetic gene circuits (e.g., SLC and Cello).

This strategy intends to address the inherent trade off in efficiency and biosafety of colonization. Building on this principle, next-generation “metabolically friendly” strains are being engineered in a rational fashion by means of GEMs. Instead of just feeding on the nutrients in the tumor, these engineered bacteria act as “metabolic partners,” they compete with tumor for critical amino acids, like asparagine, and in doing so, they rescue T cells from metabolic paralysis. In addition, with the introduction of AI-based prediction models such as SHASI-ML and DeepLigand, personalized, highly specific vaccine strategies and accurate identification of immunogenic neoantigens are becoming more feasible.

Clinically, agents such as Saltikva and SGN1 have shown extraordinary promise in combination with chemotherapy and immune checkpoint inhibitors, confirming that attenuated *Salmonella* is a viable intratumoral delivery vehicle that modulates the tumor microenvironment. These bacteria are able to reach the tumor core, an area that is not accessible by most drugs, to trigger several modalities of programmed cell death and to turn immunologically “cold tumors” into “hot tumors.” In summary, engineered attenuated *Salmonella* is an intelligent living drug platform that can self-replicate, actively target tumors, and deliver multifunctional payloads. It is anticipated that it will come to represent the fifth leg of treatment for cancer following surgery, chemoradiotherapy (CRT), targeted therapy, and immunotherapy and provide a novel means by which we may surmount the stiffest barriers in solid tumor therapy.
